# Identification of key genes in chronic intermittent hypoxia-induced lung cancer progression based on transcriptome sequencing

**DOI:** 10.1186/s12885-023-11785-3

**Published:** 2024-01-05

**Authors:** Li-Da Chen, Li Lin, Ji-Zhi Chen, Yang Song, Wei-Liang Zhang, Huang-Yu Li, Jia-Min Luo, Xiao-Bin Zhang

**Affiliations:** 1 Department of Respiratory and Critical Care Medicine, Zhangzhou Affiliated Hospital of Fujian Medical University, Zhangzhou, Fujian Province China; 2 Department of Emergency Medicine, Zhangzhou Affiliated Hospital of Fujian Medical University, Zhangzhou, Fujian Province China; 3 Ningde Food and Drug Inspection Testing Center, Ningde, Fujian Province China; 4 The School of Clinical Medicine, Fujian Medical University, No. 1, Xuefu North Road, University New District, Fuzhou, Fujian Province 350122 People’s Republic of China; 5 Department of Pulmonary and Critical Care Medicine, Zhongshan Hospital, Xiamen University, Xiamen, Fujian Province China

**Keywords:** Lung cancer, Chronic intermittent hypoxia, Obstructive sleep apnea, Transcriptome, RNA sequencing

## Abstract

**Background:**

Obstructive sleep apnea (OSA) is associated with increased risk of lung cancer mortality. Nevertheless, little is known about the underlying molecular mechanisms. This research aimed to investigate differentially expressed genes (DEGs) and explore their function in Lewis lung carcinoma (LLC)-bearing mice exposed to chronic intermittent hypoxia (CIH) by transcriptome sequencing.

**Methods:**

Lung cancer tissues in LLC-bearing mice exposed to CIH or normoxia were subjected for transcriptome sequencing to examine DEGs. Gene Ontology and Kyoto Encyclopedia of Genes and Genomes pathway analyses were employed to explore the function of DEGs. To evaluate the prognostic value of DEGs, the Kaplan–Meier survival analysis in combination with Cox proportional hazard model were applied based on The Cancer Genome Atlas.

**Results:**

A total of 388 genes with 207 up-regulated and 181 down-regulated genes were differentially expressed between the CIH and normoxia control groups. Bioinformatics analysis revealed that the DEGs were related to various signaling pathways such as chemokine signaling pathway, IL-17 signaling pathway, TGF-β signaling pathway, transcriptional misregulation in cancer, natural killer cell mediated cytotoxicity, PPAR signaling pathway. In addition, the DEGs including APOL1, ETFB, KLK8, PPP1R3G, PRL, SPTA1, PLA2G3, PCP4L1, NINJ2, MIR186, and KLRG1 were proven to be significantly correlated with poorer overall survival in lung adenocarcinoma.

**Conclusions:**

CIH caused a significant change of gene expression profiling in LLC-bearing mice. The DEGs were found to be involved in various physiological and pathological processes and correlated with poorer prognosis in lung cancer.

**Supplementary Information:**

The online version contains supplementary material available at 10.1186/s12885-023-11785-3.

## Introduction

Obstructive sleep apnea (OSA) is a highly prevalent sleep disorder characterized by intermittent partial or complete collapse, causing chronic intermittent hypoxia (CIH), sleep fragmentation, and increased inspiratory efforts. These can induce a complex series of pathophysiological changes, leading to the damage of multi-organ and multi-system. OSA has been recognized as an independent risk factor for incident cardiovascular disease [1], cognitive impairment [2], and metabolic disease [3]. Accumulating evidence shows that OSA is also independently associated with increased cancer incidence and mortality [4, 5].

Lung cancer is reported to be the most common cause of cancer mortality worldwide, with an estimated 1.8 million deaths [6]. The associations between OSA and occurrence and mortality of lung cancer were supported by more and more studies [7–11]. For instance, a cross-sectional study of 302 subjects demonstrated that sleep apnea and its related nocturnal hypoxia were independently related with an increased prevalence of lung cancer [7]. In addition, Huang et al. [8] found that severe OSA was an risk factor of cancer mortality in stage III-IV lung cancer patients. However, the mechanisms whereby OSA results in increased risks of lung cancer incidence and mortality remain unclear.

In recent decades, RNA sequencing has become an established and powerful method for analyzing differential gene expression and differential splicing of mRNAs. RNA sequencing has been used to uncover molecular mechanisms and explore potential diagnostic and therapeutic targets for many kinds of diseases. Model of CIH has generally been used so as to mimic human OSA [12]. The present study was carried out to identify differentially expressed genes (DEGs) and explore their function in Lewis lung carcinoma (LLC)-bearing mice exposed to CIH by transcriptome sequencing. In addition, the connections between different expression levels of DEGs and prognosis in lung adenocarcinoma were also investigated.

## Methods

### Animal

Seven-week-old male C57BL/6 mice were obtained from Chinese Academy of Science Laboratory Animals Center located in Shanghai. Mice were allocated to either the normoxia control (NC) (*n* = 12) or the CIH group (*n* = 12). All mice were housed in standard cages with a 12-h-day/12-h-night cycle and kept in an animal room. They were free access to water and food. This protocol was approved by the IACUC and IBC Committee in Zhongshan Hospital, Xiamen University (approved number: 2017–015) and performed according to the Guide for the Care and Use of Laboratory Animal. This study is reported in accordance with ARRIVE guidelines.

### Intermittent hypoxia (IH) exposure

Mice in CIH group were exposed to IH for 5 consecutive weeks. IH exposure was conducted in the light-on period, from 9 AM to 5 PM. The protocol of IH has been reported in our previous study [13]. The one cycle of IH consisted of the 50 s of nitrogen, lasting for 10 s, oxygen for 10 s, and subsequent compressed air for 50 s. The range of oxygen concentration was between 21% ± 1% to 6% ± 1%.

### Lung cancer cell culture and tumor induction

LLC cells were obtained from CoBioer Biosciences Co., Ltd. China. The cells were cultured in high-glucose Dulbecco's Modification of Eagle's Medium in combination with 10% fetal bovine serum (GIBCO, USA). The right flank of each mouse was injected with the LLC cells at density of 1 × 10^6^ LLC/100 μL PBS after 7 days of IH treatment. The tumor volume was recorded every 5 days after a tumor became palpable. The tumor width (W) and length (L) were collected and used for calculating the tumor volume (mm^3^) (V = W^2^ × L/2).

### Tissue preparation

All the mice were euthanized with intraperitoneal injection of pentobarbital (150 mg/kg) after 5 weeks of the IH treatment. The tumors were excised, weighted and subsequently stored in RNA locker. 6 lung cancer tissue samples were randomly selected from each group and then subjected for sequencing.

### RNA isolation, library preparation, and transcriptome sequencing

The total RNA was extracted from the lung cancer tissue using TRIzol® Reagent (Magen). Oligo(dT) magnetic beads was used for mRNA purification. Then the fragmentation was carried out with divalent cations. Subsequently, the double stranded cDNAs were synthesized. PCR amplification was performed with adaptor-ligated cDNA. Furthermore, we purified PCR products. Finally, the library preparations were sequenced on an Illumina Novaseq 6000 platform at Shanghai Applied Protein Technology Co., Ltd. Raw sequence data was deposited in the in the NCBI SRA database repository, accession number PRJNA948556.

### Data processing

Initially, we processed raw data of fastq format through in-house perl scripts. In order to obtain mapped reads, the clean reads were separately aligned to reference genome with orientation mode using HISAT2 software (http://daehwankimlab.github.io/hisat2/). We used FeatureCounts (http://subread.sourceforge.net/) to count the reads numbers mapped to each gene. Then fragments per kilobase of transcript per million mapped fragments (FPKM) of each gene was calculated based on reads count mapped to the gene and the length of this gene. Differential expression analysis was performed using the DESeq2 (http://bioconductor.org/packages/release/bioc/html/DESeq2.html). Genes with *p* value < 0.05 and | log2FC |> 1 were judged to be DEGs.

### Enrichment analysis

The Gene Ontology (GO) and Kyoto Encyclopedia of Genes and Genomes (KEGG) enrichment analysis can explain the functional enrichment of DEGs and clarify the differences between samples at the gene function level. TopGO package (http://www.bioconductor.org/packages/release/bioc/html/topGO.html) for GO function enrichment and KEGG pathway enrichment analysis (http://www.kegg.jp/kegg/kegg1.html) were used in this study. GO or KEGG function was considered to be significantly enriched when *p* value < 0.05.

### qRT-PCR

Six genes were selected to validate the sequencing data with a real-time PCR technology. Real-time PCR reactions were conducted on an ABI 7500 thermocycler (Applied Biosystems, USA). The 2-ΔΔCt method was utilized to analyze the relative gene expressions. The primer sequences are presented in Supplementary Table S1. Data were analyzed using GraphPad Prism 5.0. Data were expressed as mean ± SD. Unpaired Student’s t-test was used for comparisons of qRT-PCR data between the two groups. Statistical significance was determined as *p*-value < 0.05.

### Prognosis analysis

In order to evaluate the prognostic value of DEGs, the Kaplan–Meier survival analysis combined with Cox proportional hazard model were applied based on The Cancer Genome Atlas (TCGA) using R language packages (survival and survminer). R software (version 4.2.1) was used for all statistical analyses.

## Results

### IH promoted lung cancer growth

The final tumor weight and volume were much higher in the CIH group when compared with NC group. While CIH group mice gained less body weight. The detailed data have been published in our previous research article [13].

### Summary of transcriptomic profiles

The raw reads of each library ranged from 40, 131, 160 to 51, 498, 348. The GC content and Q30 values for all libraries were beyond 49.5% and 91.93%, respectively (Supplementary Table S2). The ratio of clean reads to raw reads was over 98%. An average 94.59% of the reads, including multiple mapped reads and unique mapped reads, were mapped to the reference genome. The results suggested that the data were reliable and eligible for the subsequent analysis. The ratio of FPKM value less than 1 is high, which revealed that the majority of genes were expressed at low levels (Supplementary Figure 1A). The values of distribution were similar among the 12 samples (Supplementary Figure 1B). A principal component analysis (PCA) was conducted to check the clustering pattern of the samples. The PC1 and PC2 were 47.23% and 12%, respectively. Although not too far from one another, the distance between CIH and NC was apparent and sufficient for the analysis (Supplementary Figure 1C).

### Identification of DEGs

**Fig. 1:**
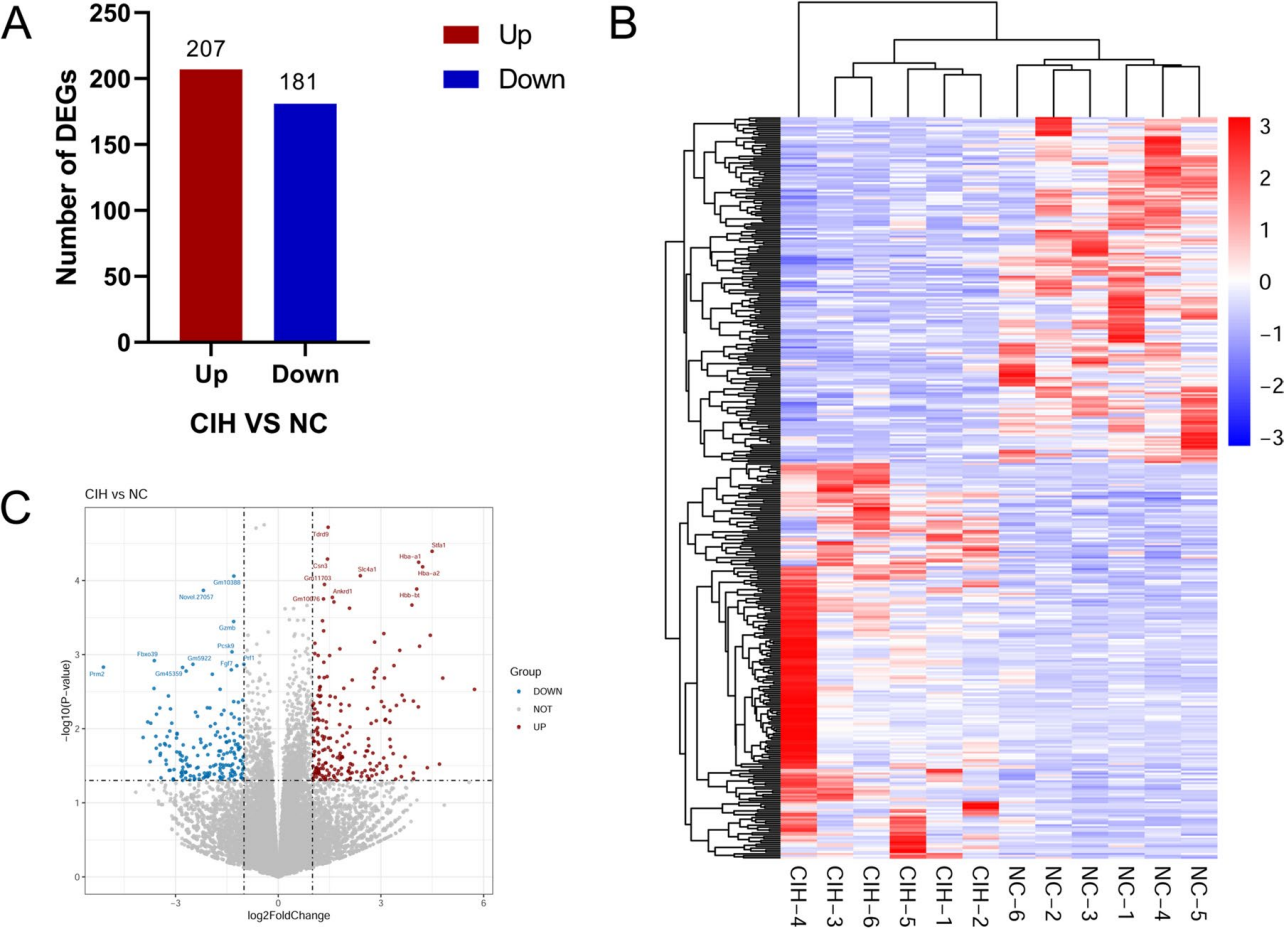
DEGs between CIH group and NC group. **A** Bar graph for the number of up- and down-regulated DEGs. **B** Heat map generated by hierarchical clustering of DEGs in CIH group and NC group, red color representing increased expressed genes and blue color for decreased expressed genes. **C** Volcano plot of DEGs between CIH group and NC group, red dots representing the up-regulated DEGs, blue dots representing the down-regulated DEGs

The results presented that there were 388 DEGs, including 207 up-regulated and 181 down-regulated genes, between the CIH group and NC group (Fig. 1A). Then we conducted hierarchical clustering analysis and revealed distinct clustering of CIH samples from NC samples (Fig. 1B). In addition, volcano plots were constructed using *p* value and fold change value to visualize differential expression between NC and CIH group (Fig. 1C). The top 20 up-regulated and down-regulated DEGs were summarized in Tables 1 and 2, respectively.

**Table 1:** List of top 20 up-regulated DEGs in CIH group as compared to controls

Gene ID	Symbol	Description	Fold change	*p*-value	Padj
ENSMUSG00000094335	Igkv1-117	immunoglobulin kappa variable 1–117	5.7327	0.002952	0.79233
ENSMUSG00000020295	Hbq1a	hemoglobin, theta 1A	4.8057	0.002078	0.72216
ENSMUSG00000071561	Cstdc5	cystatin domain containing 5	4.7103	0.030104	0.99928
ENSMUSG00000071562	Stfa1	stefin A1	4.4945	0.000040	0.28385
Novel.22907	-	-	4.4423	0.000549	0.51892
Novel.24992	-	-	4.355	0.03363	0.99928
ENSMUSG00000069917	Hba-a2	hemoglobin alpha, adult chain 2	4.218	0.000066	0.28385
ENSMUSG00000025889	Snca	synuclein, alpha	4.1291	0.000772	0.61647
ENSMUSG00000069919	Hba-a1	hemoglobin alpha, adult chain 1	4.1016	0.000057	0.28385
ENSMUSG00000091694	Apol11b	apolipoprotein L 11b	4.0931	0.005088	0.88345
ENSMUSG00000073940	Hbb-bt	hemoglobin, beta adult t chain	4.0445	0.000131	0.34395
ENSMUSG00000062563	Cys1	cystin 1	3.9502	0.047512	0.99928
ENSMUSG00000076934	Iglv1	immunoglobulin lambda variable 1	3.948	0.039583	0.99928
ENSMUSG00000023995	Tspo2	translocator protein 2	3.9469	0.004243	0.87208
ENSMUSG00000052305	Hbb-bs	hemoglobin, beta adult s chain	3.9053	0.000215	0.36598
ENSMUSG00000105906	Iglc1	immunoglobulin lambda constant 1	3.7046	0.024181	0.99928
ENSMUSG00000022902	Stfa2	stefin A2	3.6797	0.004161	0.87208
ENSMUSG00000048148	Nwd1	NACHT and WD repeat domain containing 1	3.6187	0.00353	0.81902
Novel.5311	-	-	3.5985	0.044527	0.99928
ENSMUSG00000107163	Gm43496	predicted gene 43496	3.5731	0.032045	0.99928

*Abbreviation*: *DEGs* differentially expressed genes, *CIH* chronic intermittent hypoxia

**Table 2:** List of top 20 down-regulated DEGs in CIH group as compared to controls

Gene ID	Symbol	Description	Fold change	*p*-value	Padj
ENSMUSG00000038015	Prm2	protamine 2	-5.1061	0.0014801	0.71203
ENSMUSG00000033849	B3galt2	UDP-Gal:betaGlcNAc beta 1,3-galactosyltransferase, polypeptide 2	-3.9473	0.013142	0.99928
ENSMUSG00000022383	Ppara	peroxisome proliferator activated receptor alpha	-3.8115	0.0081169	0.96581
ENSMUSG00000105226	A930028O11Rik	RIKEN cDNA A930028O11 gene	-3.7421	0.027874	0.99928
ENSMUSG00000022603	Mroh4	maestro heat-like repeat family member 4	-3.7242	0.0084388	0.97536
ENSMUSG00000043050	Tnp2	transition protein 2	-3.6288	0.0028667	0.79233
ENSMUSG00000070388	Fbxo39	F-box protein 39	-3.6198	0.0012068	0.6777
ENSMUSG00000088685	Gm23995	predicted gene, 23995	-3.5741	0.012691	0.99928
ENSMUSG00000104239	Gm37588	predicted gene, 37588	-3.5626	0.018382	0.99928
ENSMUSG00000116534	Gm49731	predicted gene, 49731	-3.5561	0.006382	0.9073
ENSMUSG00000086097	Gm16250	predicted gene 16250	-3.4607	0.0052575	0.88345
ENSMUSG00000112719	Gm45925	predicted gene, 45925	-3.4518	0.016509	0.99928
ENSMUSG00000076463	Trbv3	T cell receptor beta, variable 3	-3.4495	0.022856	0.99928
ENSMUSG00000085932	Gm15556	predicted gene 15556	-3.4409	0.021478	0.99928
ENSMUSG00000076484	Trbj1-2	T cell receptor beta joining 1–2	-3.4239	0.046047	0.99928
ENSMUSG00000108393	Gm32633	predicted gene, 32633	-3.3188	0.015919	0.99928
ENSMUSG00000030834	Abcc6	ATP-binding cassette, sub-family C (CFTR/MRP), member 6	-3.2941	0.01653	0.99928
ENSMUSG00000095078	Gm5866	predicted gene 5866	-3.2791	0.026655	0.99928
ENSMUSG00000081194	Gm8424	predicted gene 8424	-3.2697	0.030174	0.99928
ENSMUSG00000103591	Gm38365	predicted gene, 38365	-3.2455	0.012807	0.99928

*Abbreviation*: *DEGs* differentially expressed genes, *CIH* chronic intermittent hypoxia

### GO enrichment and KEGG pathway analyses

**Fig. 2:**
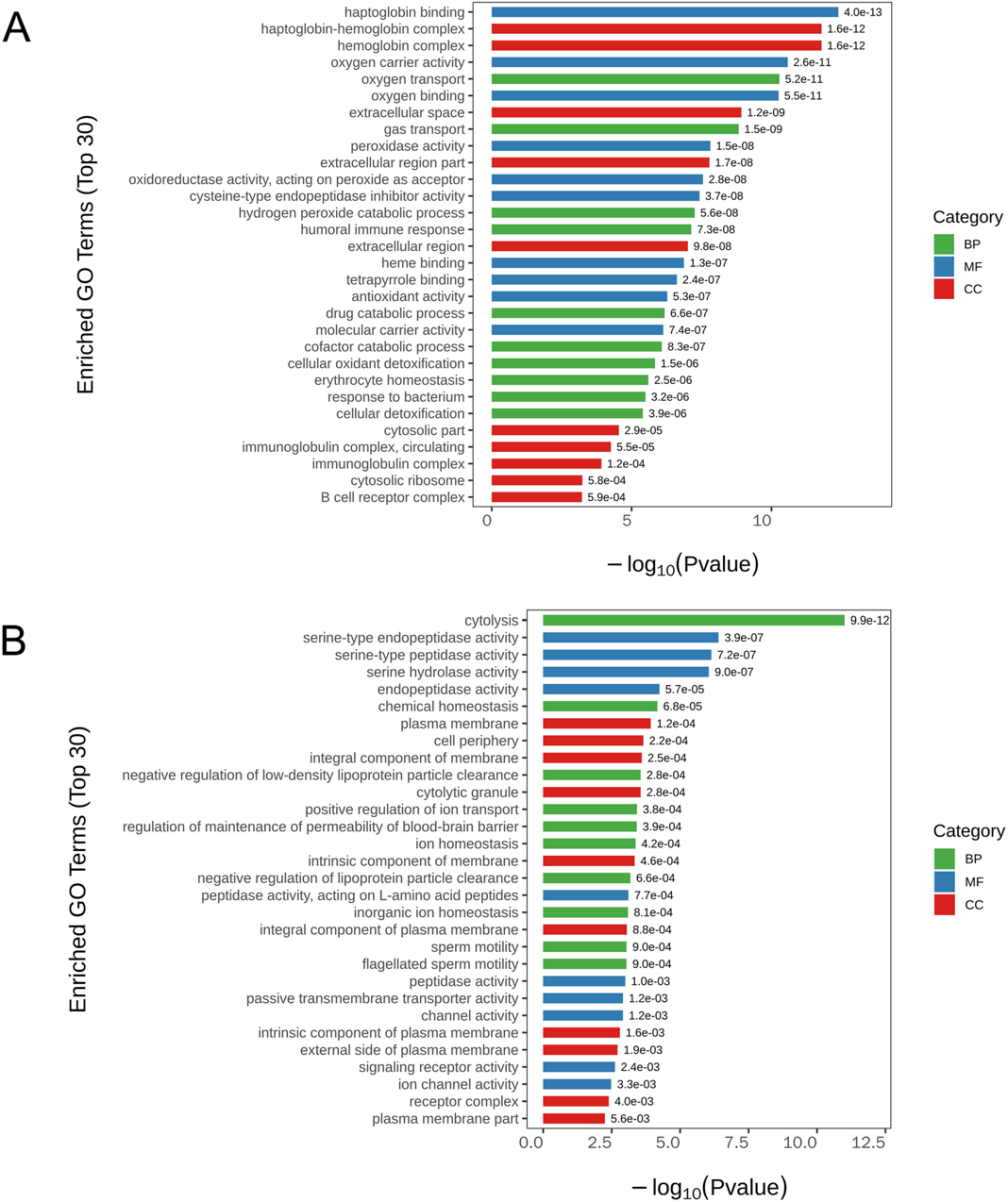
GO analyses of DEGs. **A** GO terms for the up-regulated DEGs. **B** GO terms for the down-regulated DEGs. Y-axis represents GO terms, X-axis represents the *p* value

**Fig. 3:**
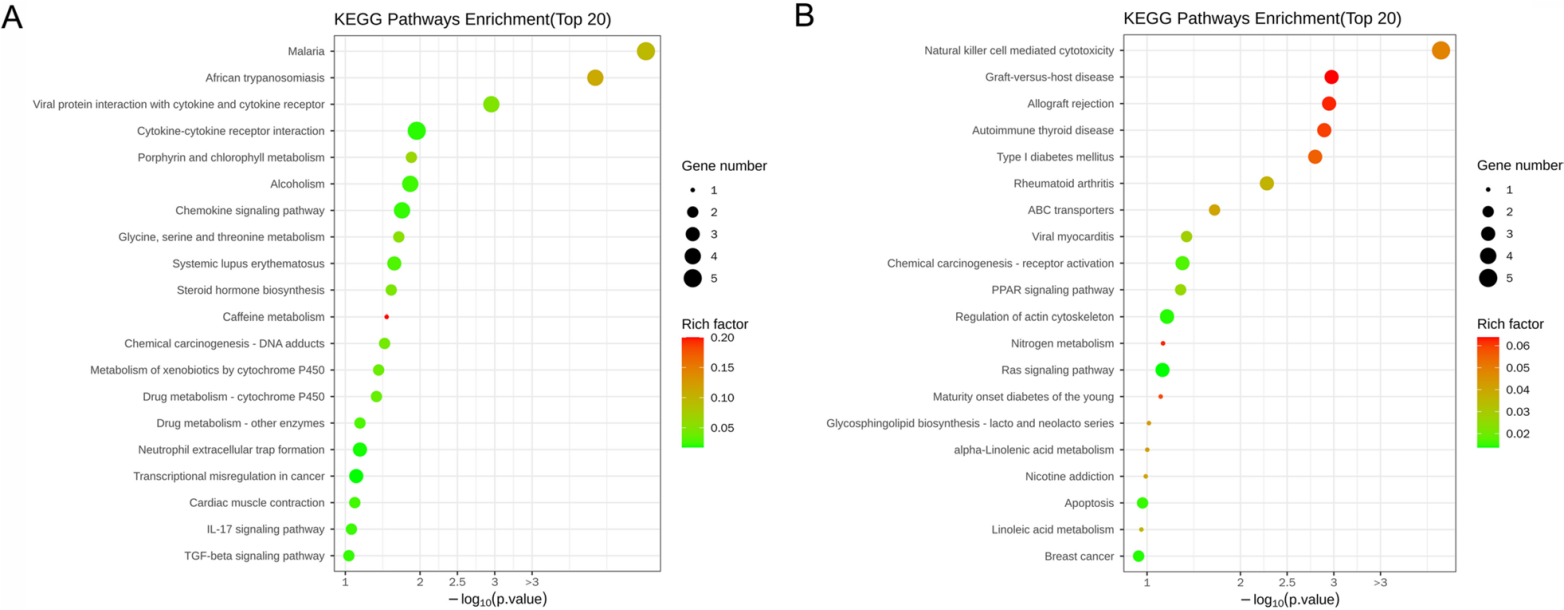
KEGG pathway analyses of DEGs. **A** Top 20 pathways associated with up-regulated DEGs (**B**) Top 20 pathways related to down-regulated DEGs. The size of the dot represents the number of genes, the color of the dot represents risk factor, Y-axis shows pathway name, X-axis shows the *p* value

To better reveal the function of these DEGs, GO function enrichment and KEGG pathway enrichment analyses were conducted. The top 10 GO terms including biological process, molecular function, and cell component for up-regulated and down-regulated DEGs were displayed in Fig. 2A and B, respectively. We found that these DEGs were involved in various molecular functions and biological processes. The up-regulated DEGs were mainly enriched in pathways such as IL-17 signaling pathway, TGF-β signaling pathway, chemokine signaling pathway, transcriptional misregulation in cancer (Fig. 3A). The main KEGG pathways for down-regulated DEGs were natural killer cell mediated cytotoxicity, ABC transporters, and PPAR signaling pathway, among others (Fig. 3B).

### Prognosis analysis

**Fig. 4:**
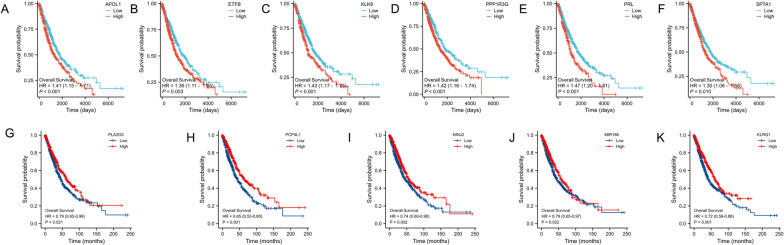
The overall survivals of lung adenocarcinoma patients with high or low gene expression. **A**-**F** The up-regulated DEGs. (G-K) The low-regulated DEGs

The prognostic value of the DEGs in lung adenocarcinoma patients was examined using Kaplan–Meier survival curves based on TCGA datasets. Univariate Cox regression analysis suggested that the high expression levels of up-regulated DEGs including APOL1, ETFB, KLK8, PPP1R3G, PRL, and SPTA1 were significantly correlated with poorer overall survival (OS) in lung adenocarcinoma. The low expression levels of down-regulated DEGs PLA2G3, PCP4L1, NINJ2, MIR186, and KLRG1 were also statistically correlated with a shorter OS in lung adenocarcinoma (Fig. 4).

### Validation by qRT-PCR

**Fig. 5:**
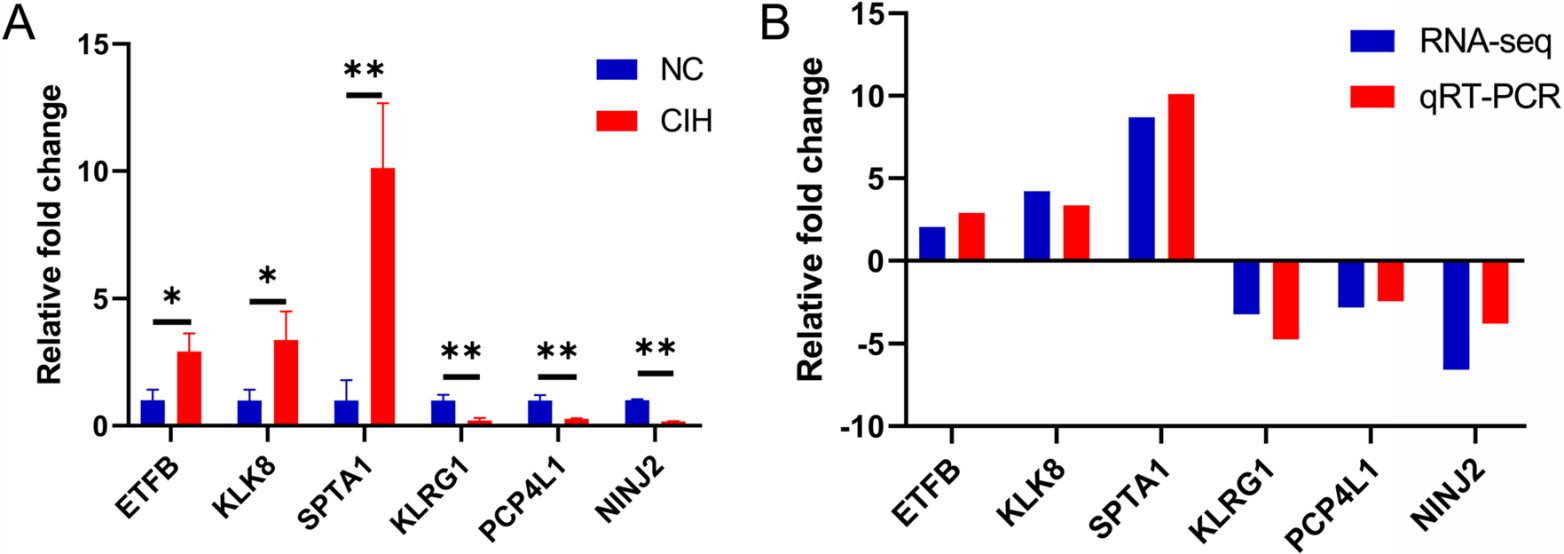
DEGs validated by qRT-PCR. **A** The relative expression levels of 6 DGEs by qRT-PCR analysis (*n* = 3). **B** The expression pattern of DEGs in both qRT-PCR and RNA-seq. *p < 0.05; **p < 0.01

To verify the accuracy of the RNA sequencing data, 6 prognosis-related genes including 3 up-regulated and 3 down-regulated genes were selected for validation experiments. The results indicated that the expression levels of ETFB, KLK8, and SPTA1 were remarkably up-regulated, and the expression levels of KLRG1, PCP4L1 and NINJ2 were significantly down-regulated (Fig. 5A). This was consistent with sequencing data, confirming the accuracy of sequencing (Fig. 5B).

## Discussion

This study established models of CIH and lung cancer in mice to explore the potential mechanisms whereby OSA promoted lung cancer progression. 388 genes were differentially expressed in lung cancer between the CIH-treated LLC-bearing mice and NC group. Further bioinformatics analysis found that these DEGs were related to various pathways involving chemokine signaling pathway, IL-17 signaling pathway, TGF-β signaling pathway, transcriptional misregulation in cancer, natural killer cell mediated cytotoxicity, PPAR signaling pathway. In addition, we identified 11 DEGs, which were associated with unfavorable prognosis in lung adenocarcinoma patients.

Growing evidence suggests a close relationship between OSA and cancer incidence and mortality [4, 5]. A community-based cohort including 400 residents with 20 years follow-up reported that moderate-to-severe OSA was independently correlated with an increased risk of cancer incidence and mortality [14]. Nieto et al. [15] demonstrated a dose–response relationship between OSA and cancer mortality in a community-based sample. A multicenter study with 5427 patients showed that OSA severity was associated with increased cancer mortality after follow-up of 4.5 years [16]. As for lung cancer type, a prospective study followed up 65,330 women and suggested that no independent association existed between OSA and overall cancer risk. However, significant associations were noted for the smoking-related cancers (e.g., lung cancer) [11]. A meta-analysis revealed that OSA patients had an nearly 30% higher risk of lung cancer compared with non-OSA subjects after pooling data of four observational studies with 4,885,518 patients [9]. Liu et al. [10] showed that the recurrence rate, metastasis rate and mortality increased in lung cancer patients with OSA during the one-year follow-up period. Li et al. [17] also demonstrated an association between OSA and increased risk for mortality in lung cancer patients. Furthermore, they reveal some molecular pathways involved in sustained hypoxia-induced lung cancer progression some based on Gene Expression Omnibus data base. However, the model of sustained hypoxia did not reflect the pathophysiology of OSA.

These findings have been further supported by animal experiments. In a mouse model of sleep apnea, Almendros and colleagues [18] found that CIH induced a remarkable increase in melanoma lung metastasis when compared to NC group. Another study also demonstrated that CIH promoted lung epithelial TC1 cell tumor growth and invasion toward adjacent tissues in a mouse model of OSA [19]. In addition, our previous animal study showed that CIH accelerated lung cancer development and enhanced the vascular endothelial growth factor expression. The mean SUVmax values assessed by micro-PET–CT were considerably higher in the CIH group than the NC group [20]. Collectively, these results indicated that CIH was an important component of tumor malignant properties, facilitating cancer growth, invasion and migration.

The contributing mechanisms underlying this association are not yet well understood. To gain a better understanding of the mechanisms, Illumina high-throughput technology was utilized to sequence the transcriptome of LLC-bearing mice exposed to CIH. The present study revealed that the up-regulated DEGs were markedly related to chemokine signaling pathway, IL-17 signaling pathway, TGF-β signaling pathway, transcriptional misregulation in cancer. Considerable evidence linked IL-17 with lung cancer. Numasaki et al. [21] demonstrated that IL-17 obviously increased angiogenesis and promoted the growth of lung cancer transplanted in severe combined immunodeficient mice. Another study reported that intraperitoneal injection of IL-17 resulted in remarkably larger tumors in a LLC mouse model when compared with the control group [22]. Furthermore, It was reported that TGF-β could promote tumor progression through suppression of immune surveillance, angiopoiesis, and promotion of epithelial to mesenchymal transition [23, 24]. An increasing body of evidence suggested that the TGF-β pathway activation contributed to poor prognosis in lung cancer patients [25, 26]. The down-regulated DEGs were enriched in natural killer cell mediated cytotoxicity and PPAR signaling pathway. In fact, the killing mediated by natural killer cells and cytotoxic T lymphocytes represents a crucial mechanism in the immune defense against cancers. And the impairment of natural killer cell mediated cytotoxicity facilitates the growth of lung cancer. A clinical study found that severe OSA had considerably fewer invariant natural killer T cells (iNKT) compared to mild-moderate OSA or no OSA patients and 12 months of continuous positive airway pressure therapy increased the frequency of iNKT cells. Furthermore, they found that hypoxia resulted in impaired cytotoxicity [27].

The present study also revealed several DEGs related to poorer prognosis in lung adenocarcinoma patients. Planque et al. [28] analyzed KLK8 mRNAs in 60 NSCLC tissues and in paired unaffected tissues by PCR and found that KLK8-T4 alternative splice variant, alone or in combination was independent marker of poor prognosis in lung cancer. Another study demonstrated that MIR186 could inhibit lung cancer progression through targeting SIRT6 [29]. In addition, a study showed that the deficiency of PRMT5 enhanced Klrg1^+^ terminal CD8^+^ T cell development and eliminated antitumor activity [30]. So this indicated that the DEGs identified in this study could be promising candidates for future research in investigating the association between OSA and increased cancer mortality. The identified DEGs collectively suggest a molecular landscape favoring tumor growth, immune evasion, and inflammatory responses in the context of CIH-induced lung cancer. The dysregulation of pathways related to angiogenesis, immune modulation, and transcriptional control may contribute to the observed poorer prognosis in lung adenocarcinoma.

The current study has some strengths. Firstly, high-throughput sequencing was utilized to investigate the mechanisms linking OSA with progression of lung cancer. Secondly, the findings of the key signaling pathways and prognosis related genes provided a new direction for future study on this issue. Thirdly, the lung cancer prognosis related genes were validated in both animal model and human study, which made the result more reliable. Our study also has several limitations that warrant mention. Firstly, the CIH animal models only simulated one of the major features of OSA and lacked of other features (e.g., hypercapnia and sympathetic hyperactivity). Secondly, although the DEGs and their potential function were identified and analyzed, the functional and mechanistic study on cell line were not conducted. Finally, only LLC-bearing mouse model was established. Whether these results can also be successfully applied to other lung cancer type needed further confirmation. Recognizing the heterogeneity of lung cancer, future studies should incorporate different lung cancer subtypes, such as squamous cell carcinoma and small cell carcinoma.

## Conclusions

CIH caused a significant change of gene expression profiling in LLC-bearing mice. The dysregulated genes were involved in chemokine signaling pathway, IL-17 signaling pathway, TGF-β signaling pathway, transcriptional misregulation in cancer, natural killer cell mediated cytotoxicity, PPAR signaling pathway. Meanwhile, 11 DEGs were identified to be associated with poorer prognosis in lung adenocarcinoma. The collective impact of these DEGs on key pathways suggested a multi-faceted contribution to CIH-induced lung cancer progression, providing potential insights into the mechanisms underlying the observed poorer prognosis of lung adenocarcinoma in OSA patients.

### Supplementary Information

**Additional file 1:** **Supplementary Figure 1.** Summary of transcriptomic profiles. (A) The percentage of different FPKM ranges in 12 samples. (B) Boxplot of FPKM distribution among the two groups. (C) Clustering pattern of the samples examined by PCA.

**Additional file 2:** **Supplementary Table S1.** Primers used for qRT-PCR.

**Additional file 3:** **Supplementary Table S2.** Quality control.

## Data Availability

The data presented in the study are deposited in the NCBI SRA database repository, accession number PRJNA948556.
