# Supplementary material for: Identification of key genes in chronic intermittent hypoxia-induced lung cancer progression based on transcriptome sequencing
Source: BMC Cancer. 2024 Jan 5;24:41. doi: 10.1186/s12885-023-11785-3 (PMC10770984; doi:10.1186/s12885-023-11785-3)

Supplementary Figure 1 Summary of transcriptomic profiles. (A) The percentage of different FPKM ranges in 12 samples. (B) Boxplot of FPKM distribution among the three groups. (C) Clustering pattern of the samples examined by PCA.


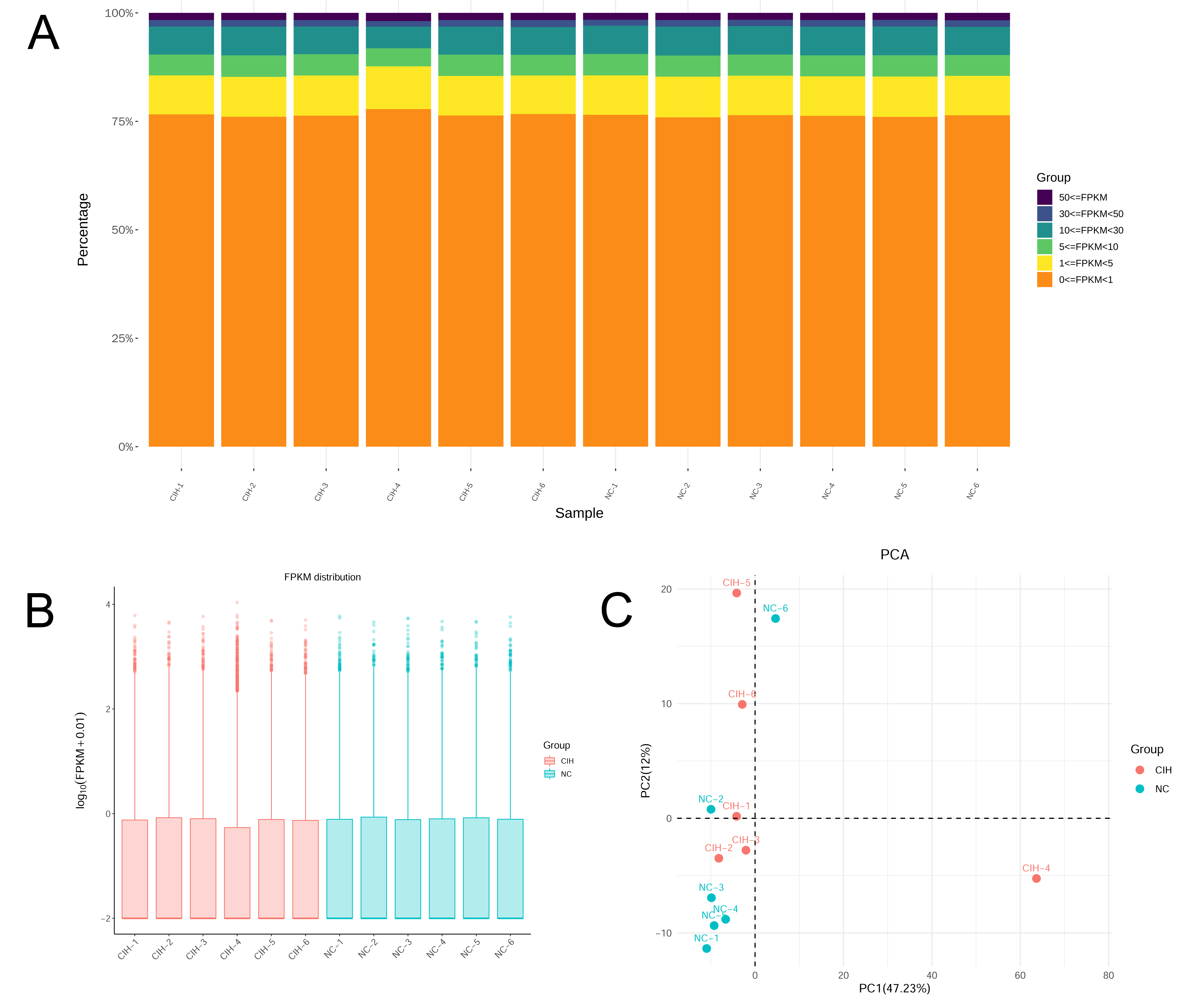

Supplement: Supplementary file 1 — Additional file 1: Supplementary Figure 1. Summary of transcriptomic profiles. (A) The percentage of different FPKM ranges in 12 samples. (B) Boxplot of FPKM distribution among the two groups. (C) Clustering pattern of the samples examined by PCA. [file 12885_2023_11785_MOESM1_ESM.docx]
