# Supplementary material for: Identification of key genes in chronic intermittent hypoxia-induced lung cancer progression based on transcriptome sequencing
Source: BMC Cancer. 2024 Jan 5;24:41. doi: 10.1186/s12885-023-11785-3 (PMC10770984; doi:10.1186/s12885-023-11785-3)
Supplement: Supplementary file 2 — Additional file 2: Supplementary Table S1. Primers used for qRT-PCR. [file 12885_2023_11785_MOESM2_ESM.docx]

Supplementary Table S1 Primers used for qRT-PCR.

| Genes | forward primer | reverse primer |
| --- | --- | --- |
| β-actin | 5′-GTACCACCATGTACCCAGGC-3′ | 5′-AACGCAGCTCAGTAACAGTCC-3′ |
| ENSMUSG00000004610 | 5′-AGGCTATTGATGATGACTGTAACCA-3′ | 5′-TGTCACCTGAGAGGCGAATGTA-3′ |
| ENSMUSG00000064023 | 5′-GCCAATCTGTGTCCCAAAGT-3′ | 5′-GAATAGATTTTCACTTCCGCAC-3′ |
| ENSMUSG00000026532 | 5′-GGATGCGAATGCAACACAAT-3′ | 5′-GAGCGGAACTCTTTGTGAGT-3′ |
| ENSMUSG00000030114 | 5′-TGCAGACAAAGGCTCACATC-3′ | 5′-TGACCCAGGTACTCTCCAAA-3′ |
| ENSMUSG00000038370 | 5′-AAACACCTCCAGCAGCCAAC-3′ | 5′-GGGAGAGAGGAAGTAGAAGGGTT-3′ |
| ENSMUSG00000041377 | 5′-TAGCCTCCTTCTTGTGTTCATC-3′ | 5′-ACAAAGGCTGAAGTGGCTCTA-3′ |
