# Supplementary material for: Identification of key genes in chronic intermittent hypoxia-induced lung cancer progression based on transcriptome sequencing
Source: BMC Cancer. 2024 Jan 5;24:41. doi: 10.1186/s12885-023-11785-3 (PMC10770984; doi:10.1186/s12885-023-11785-3)
Supplement: Supplementary file 3 — Additional file 3: Supplementary Table S2. Quality control. [file 12885_2023_11785_MOESM3_ESM.docx]

Supplementary Table S2 Quality control

| Sample | Raw reads | Clean reads | Clean bases | Error(%) | Q20(%) | Q30(%) | GC(%) |
| --- | --- | --- | --- | --- | --- | --- | --- |
| CIH-1 | 46277546 | 45703164 | 6.8G | 0.03 | 97.02 | 92.07 | 50.45 |
| CIH-2 | 46115476 | 45199262 | 6.74G | 0.03 | 97.05 | 92.16 | 50.08 |
| CIH-3 | 44725754 | 43928100 | 6.55G | 0.03 | 97.14 | 92.32 | 50.8 |
| CIH-4 | 51498348 | 51232588 | 7.66G | 0.03 | 98.02 | 94.03 | 49.96 |
| CIH-5 | 44110374 | 43454036 | 6.47G | 0.03 | 97.18 | 92.4 | 50.23 |
| CIH-6 | 43377264 | 42687916 | 6.34G | 0.03 | 97.11 | 92.27 | 50.79 |
| NC-1 | 46272062 | 45592106 | 6.8G | 0.03 | 97.15 | 92.31 | 49.5 |
| NC-2 | 44863350 | 44394892 | 6.61G | 0.03 | 97.2 | 92.4 | 50.67 |
| NC-3 | 41118076 | 40785072 | 6.08G | 0.03 | 97.25 | 92.45 | 49.58 |
| NC-4 | 40131160 | 39730168 | 5.92G | 0.03 | 97.01 | 91.93 | 50.17 |
| NC-5 | 45069124 | 44242186 | 6.58G | 0.03 | 97.17 | 92.44 | 50.13 |
| NC-6 | 45600166 | 44957548 | 6.71G | 0.03 | 97.26 | 92.58 | 49.88 |
